# Rare Mutations in CCDC7 Contribute to Early-Onset Preeclampsia by Inhibiting Trophoblast Migration and Invasion

**DOI:** 10.3390/jpm14030253

**Published:** 2024-02-27

**Authors:** Hu Tan, Li Yu, Jingsi Chen, Xiaoyi Wang, Fang He, Lin Yu, Lili Du, Dunjin Chen

**Affiliations:** 1 Key Laboratory for Major Obstetric Diseases of Guangdong Province, Department of Obstetrics and Gynecology, The Third Affiliated Hospital of Guangzhou Medical University, Guangzhou 510150, China; 2 The Medical Centre for Critical Pregnant Women in Guangzhou, Guangzhou 510150, China; 3 Obstetrics & Gynecology Institute of Guangzhou, The Third Affiliated Hospital of Guangzhou Medical University, Guangzhou 510150, China; 4 Department of Fetal Medicine and Prenatal Diagnosis, The Third Affiliated Hospital of Guangzhou Medical University, Guangzhou 510150, China; 5 Guangdong-Hong Kong-Macao Greater Bay Area Higher Education Joint Laboratory of Maternal-Fetal Medicine, Guangzhou 510150, China; 6 Guangdong Engineering and Technology Research Center of Maternal-Fetal Medicine, Guangzhou 510150, China

**Keywords:** early-onset preeclampsia, CCDC7, rare variants, trophoblast, migration and invasion

## Abstract

Rare gene variants have been found to play a role in complex disorders. Preeclampsia, and especially early-onset preeclampsia, has a strong genetic link. However, the role of rare variants in the offspring of mothers with preeclampsia remains unclear. In this study, whole-exome sequencing (WES) was used to identify rare pathogenic variants in two families with early-onset preeclampsia. Two heterozygous rare variants in *CCDC7*, c.625C>T (p.R209C) and c.1015C>T (p.R339X), were detected in two families and were cosegregated in the offspring of preeclamptic pregnancies. We examined the spatiotemporal expression pattern of *CCDC7* in human placental villi and the effects of *CCDC7* on migration and invasion of trophoblast cells JEG-3. The quantitative real-time PCR and Western blot results showed that the expression of *CCDC7* in placental villi was the lowest during the first trimester and increased as the pregnancy progressed. The *CCDC7* p.R339X variant showed a decrease in mRNA and protein expressions. Loss-of-function assays showed that knockdown of *CCDC7* suppressed the migration and invasion of JEG-3 cells. In conclusion, *CCDC7* is a potential susceptibility gene for preeclampsia, which is key for the migration and invasion of trophoblast cells. Rare variants of preeclampsia in offspring may play a crucial role in the pathogenesis of preeclampsia and require further research.

## 1. Introduction

Preeclampsia is a complex, heterogenous, pregnancy-associated disorder characterized by high blood pressure (≥140/90 mmHg) and proteinuria (≥300 mg/L per 24 h) after the 20th week of pregnancy [1]. As a leading complication of pregnancy, preeclampsia affects an estimated 4 million women each year and causes the deaths of more than 70,000 women and 500,000 newborns worldwide [2]. Preeclampsia can be divided into two groups according to the onset time: early-onset preeclampsia (EOPE, <34 weeks) and late-onset preeclampsia (LOPE, ≥34 weeks) [1]. Preeclampsia, particularly EOPE, is considered to be heritable, with an estimated heritability of about 0.50 in several populations [3,4]. Maternal and fetal contributions are estimated at 35% and 20%, respectively [5,6].

Maternal contributions to preeclampsia have attracted considerable attention over the past decades. Using genome-wide association studies (GWASs), gene variants have been reported to be associated with a high risk of preeclampsia, but most of these variants have not been replicated in different groups [7]. Some studies researched variants in the fetal genome. In multiplex Dutch preeclampsia families, variant p.Y153H of *STOX1* was cosegregated in offspring from preeclamptic pregnancies [8]. This variant resulted in reduced trophoblast invasion and played a crucial role in early utero-placental development [9,10], although the association between this variant and preeclampsia was not duplicated in two different groups [11,12]. Recently, McGinnis et al. discovered a significant susceptibility locus near the *FLT1* gene (rs4769613, *p* = 5.4 × 10^−11^) between 4380 offspring from preeclamptic pregnancies and 310,238 controls [13]. These studies have noted the importance of variants of preeclampsia offspring in the pathogenesis of preeclampsia.

With the wide application of whole-exome sequencing (WES) and whole-genome sequencing (WGS), rare variants of complex disorders, such as autism spectrum disorder [14], migraine [15], and advanced age-related macular degeneration [16], have attracted great attention. Recently, several studies have revealed the effect of rare variants on pregnancy-associated diseases. Huusko et al. detected rare and likely damaging variants in *HSPA1L* in multiple families with recurrent spontaneous preterm births [17]. Liu et al. found that novel rare variants in ABC transporter genes were associated with intrahepatic cholestasis of pregnancy disease based on a case–control study [18]. However, far too little attention has been paid to the role of rare variants in the offspring in the pathogenesis of preeclampsia.

In this study, we identified rare variants in two families with EOPE using WES and constructed mutant cell lines to elucidate the effects of the identified variants on the function of trophoblast cells.

## 2. Methods

### 2.1. Patients

**Figure 1:**
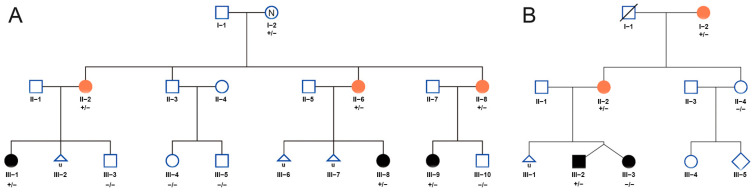
Family pedigree of people ((**A**), family A; (**B**), family B) enrolled in this study. Orange circles represent the preeclamptic mother, and black circles and boxes represent preeclamptic offspring. “+” and “−” indicate mutant allele and wild-type allele, respectively.

Two Han preeclampsia families were enrolled (Figure 1) in this study. The diagnosis of preeclampsia was based on the clinical features, including onset time at pregnancy, high blood pressure, proteinuria, impaired liver function, renal insufficiency, thrombocytopenia, pulmonary edema, headache, and visual symptoms, according to the ACOG practice bulletin No. 202 [1]. The onset time of all the women was after 20 weeks’ and before 34 weeks’ gestation, except that the medical record of the pregnancy of subject I-2 in family A was unknown and subject I-2 in family B only presented gestational hypertension. Subject I-2 in family A had developed hypertension at the last visit. The peripheral blood was collected from the women with preeclampsia and their relatives. Placental samples near the cord insertion site (8–10 pieces, size about 0.5 × 0.5 × 0.5 cm each) and cord blood (5–10 L) were collected within 10 min after delivery and were stored in RNAlateror liquid nitrogen for future analysis. This study was approved by the ethics committee of the Third Affiliated Hospital of Guangzhou Medical University. All of the participants enrolled in the study gave informed written consent.

### 2.2. Whole-Exome Sequencing and Data Analysis

Genomic DNA was extracted from placental samples, preeclampsia maternal peripheral blood, and offspring cord blood from all cases using a DNeasy Blood and Tissue Kit (Qiagen, Hilden, Germany), according to the manufacturer’s standard procedure. WES was conducted using the peripheral blood from women with preeclampsia and their relatives (cord blood was used in three newborns, III-8 in family A and III-2 and III-3 in family B, instead of peripheral blood) using next-generation sequencing platforms by iGeneTech Co., Ltd. (Beijing, China). Sequenced reads were aligned to the human reference genome GRCh37/hg19. Software including Samtools 1.9and GATK 4.0 were used to call, sort, and index variants from aligned sequence files. Annotation of genetic variants was performed using ANNOVAR. Potential maternal DNA contamination was excluded via the genotyping of short tandem repeats (STRs). The detailed information is provided in the Supplementary Materials.

This study focused on rare variants. The variants selected for downstream analysis met the following criteria: (1) minor allele frequency in population database was <0.05; (2) more than two in silico applications (Polyphen2, SIFT, or CADD) classified the variant as damaging, or it received a score ≥ 15; (3) it was cosegregated in all preeclampsia offspring from the same family. Variants selected for downstream analysis were validated via Sanger sequencing in both placental samples and offspring cord blood.

### 2.3. Cell Culture

The human placental choriocarcinoma JEG-3 cell line (ATCC: HTB-36) was cultured in Dulbecco’s modified Eagle medium: Nutrient Mixture F-12 (DMEM/F12) (Gibco, Grand Island, NY, USA), supplemented with 10% (*v*/*v*) fetal bovine serum (FBS; Gibco, Grand Island, NY, USA). Cells were maintained at 37 °C in an atmosphere containing 5% CO_2_.

### 2.4. Generation of Stable CCDC7 Knockdown Cell Lines

Lentiviral-based shRNA vectors and non-targeting shRNA vectors (containing puromycin and enhanced green fluorescent protein) were designed and synthesized by Genecopoeia (Guangzhou, China). The shRNA targeted sequences were as follows: *CCDC7* shRNA1, 5′-CCAGTAAAGCATCTGTTGACC-3′; *CCDC7* shRNA2, 5′-GCTTCGCGCCGTAGTCTTA-3′; and scrambled control, 5′-GCTTCGCGCCGTAGTCTTA-3′. Recombinant lentiviral particles were produced by co-transfecting 293Ta cells with a lentiviral expression plasmid and a Lenti-Pac™ HIV Expression Packaging Kit (GeneCopoeia, Guangzhou, China). The viral supernatants were harvested 48 h post-transfection. The JEG-3 cells were transduced with lentiviral particles in the presence of 5 μg/mL polybrene. After 48 h, the infected cells were selected with 2 μg/mL puromycin.

### 2.5. RNA Extraction and Quantitative Real-Time Polymerase Chain Reaction

Total RNA from the placenta samples was extracted using TRIzol reagent (Invitrogen, Carlsbad, CA, USA) following the manufacturer’s instructions. The synthesis of complementary DNA (cDNA) was performed by using a PrimeScript™ RT reagent Kit with gDNA Eraser (Takara, Tokyo, Japan). Quantitative real-time PCR (qRT-PCR) was conducted using a TB Green^®^ Premix Ex Taq™ II Kit (Takara, Tokyo, Japan). GAPDH mRNA levels were used as the internal control, and relative gene expression levels were calculated using the comparative Ct method (2^−ΔΔCT^). The experiments were repeated at least three times in each placental sample, and 3–5 pieces from one placenta were used.

### 2.6. Protein Extraction and Western Blot Analysis

Total protein was extracted with RIPA lysis buffer supplemented with protease and phosphatase inhibitors (Sigma-Aldrich, St. Louis, MO, USA). The protein concentrations were measured with a BCA protein assay kit (Thermo Fisher Scientific, Waltham, MA, USA). After boiling for 10 min at 95 °C in Laemmli sample buffer (BioRad Laboratories, Hercules, CA, USA), equal amounts of protein (50 μg) were separated using sodium dodecyl sulfate-polyacrylamide gel-electrophoresis (SDS-PAGE) and then transferred to polyvinylidene fluoride (PVDF) membranes (Millipore, Billerica, MA, USA). The membranes were blocked for 1 hour at room temperature with 5% nonfat dry milk in phosphate-buffered saline (PBS) containing 0.1% Tween 20 (PBST) and probed with diluted primary antibody (anti-CCDC7, Abcam, Cambridge, MA, USA; 1:1000) at 4 °C overnight. Subsequently, the membranes were washed three times with PBST and incubated with a horseradish peroxidase-conjugated secondary antibody for 1 h at room temperature. The protein bands were visualized using enhanced chemiluminescence (Thermo Scientific, Waltham, MA, USA) and Image Lab V.6.1 software (BioRad Laboratories, Hercules, CA, USA).

### 2.7. Immunofluorescence

Sections of paraffin-embedded placental tissues (5 μm thickness) were deparaffinized and rehydrated, followed by microwave antigen retrieval in citrate buffer (pH 6.0). Then, sections were incubated in 3% H_2_O_2_ for 10 min at room temperature to remove endogenous peroxidase. and washed three times in PBS. To block nonspecific antibody binding, the sections were incubated with 10% normal goat serum for 30 min at room temperature. After overnight probing with antibody specific for CCDC7 (1:100; Novus Biologicals, Littleton, CO, USA) at 4 °C, the sections were washed in PBS and incubated in the dark with Alexa Fluor 546-conjugated antirabbit IgG (1:200; Invitrogen, Carlsbad, CA, USA) for 1 h at room temperature. The nuclei were stained with DAPI (Sigma Aldrich, St. Louis, MO, USA), and the sections were mounted in fluorescent mounting medium (Dako, Carpinteria, CA, USA). Images were captured with an inverted fluorescence microscope (Nikon, Melville, NY, USA).

### 2.8. Wound-Healing Assay

After a 24 h culture, the cells were grown to 80–90% confluence. The cell monolayers were scratched with a 200 μL pipette tip in a straight line to generate a wound-like gap, followed by washing with PBS to remove the cellular debris. The cells were rephotographed after 24 h for analysis of wound closure, as previously described [19]. The cell migration rate was calculated according to the formula: [(0 h wound width − 24 h wound width)/0 h wound width] × 100%. The experiments were repeated at least three times.

### 2.9. Transwell Invasion Assay

For the transwell invasion assay, the transwell chambers (8 µm pore size, Corning Costar, NY, USA) were coated with diluted Matrigel (300 μg/mL, BD Biosciences, Franklin Lakes, NJ, USA). The transfected cells were suspended in a serum-free medium and added to the upper chambers, and the lower chambers were filled with a medium containing 10% FBS as a chemoattractant. After incubation for 24 h at 37 °C with 5% CO_2_, the invasive cells in the lower chambers were fixed with 4% paraformaldehyde for 25 min and stained with 0.1% crystal violet for 15 min. Finally, the invaded cells in five random visual fields were imaged and counted under an inverted light microscope.

### 2.10. Statistical Analysis

Results are presented as means ± SEM. A two-way Student’s *t*-test was used to determine changes in mean gene expression. For analysis of demographic data, Student’s *t*-test, χ2, or Fisher’s exact test was used to determine significant differences in the cohorts. All statistical analyses were performed using Prism 7 software (GraphPad Software, La Jolla, CA, USA), and results were considered significant when *p* < 0.05.

## 3. Results

### 3.1. Clinical Features and Rare Variants of CCDC7 Detected in Two EOPE Families

Clinical data and blood samples were collected from two nonconsanguineous Chinese families with early-onset preeclampsia. The preeclampsia pedigrees are shown in Figure 1A,B. The mean gestational age of the subjects was 28.6 ± 5.5 years. The mean onset time was 30.3 ± 4.2 weeks of gestation. The mean time of delivery was 33.4 ± 3.4 weeks of gestation. Most of subjects presented with EOPE, except for two subjects: II-8 in family A, who had eclampsia at 32 weeks of gestation, and I-2 in family B, who presented with high blood pressure at 36 weeks of gestation but the information on the onset time of high blood pressure and other symptoms was unknown due to a lack of medical records. At the last visit, I-2 in family A presented hypertension, and other pregnant women did not develop hypertension disorders after delivery, which needed a long-term follow-up since they were young. The detailed features of the enrolled preeclampsia subjects are shown in Table 1.

### 3.2. Whole-Exome Sequencing Identified Rare Variants in CCDC7 in Familial Preeclampsia

**Figure 2:**
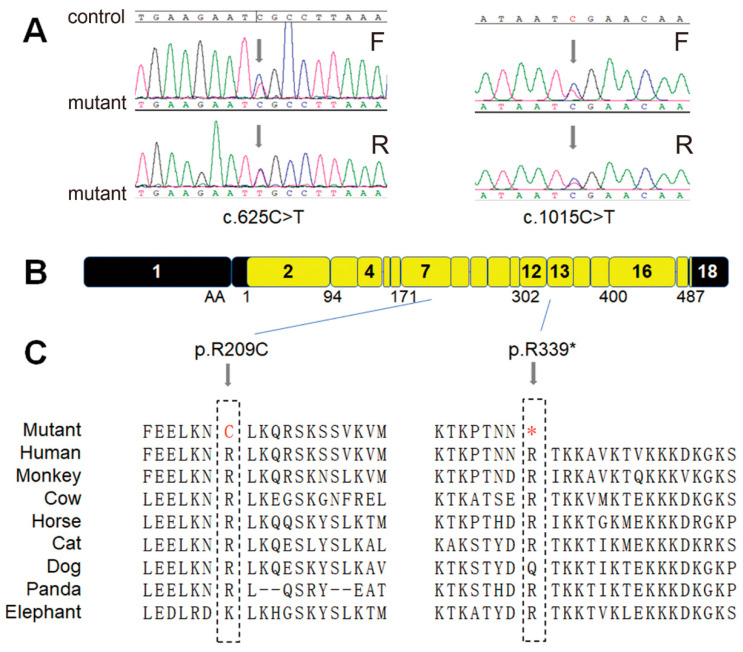
Rare variants of CCDC7 detected in two EOPE families and conservation analysis. (**A**) Variants c.625C>T (p.R209C) and c.1015C>T (p.R339*) were identified in family A and family B, respectively; (**B**) variants c.625C>T (p.R209C) and c.1015C>T (p.R339*) localized in exon 7 and exon 13, respectively; and (**C**) both of these two mutations are located in highly conservational sites across multiple species. Red represents the mutant site and “*” indicates a stop codon.

The genomic DNA of the patients and their families were sent for WES analysis, and bioinformatics analysis were performed to explore the potential pathogenic variants in an unbiased way. We detected two heterozygous rare variants (c.625C>T/p.R209C and c.1015C>T/p.R339*) in the *CCDC7* (NM_145023.6) gene in patients and their affected children. Then, the variants were confirmed via Sanger sequencing (Figure 2A). Both of these mutant sites were highly conserved in multiple mammalian species (Figure 2B,C). The missense variant (p.R209C) is predicted to be damaging, according to online software SIFT, Polyphen2, and CADD (score ≥ 15). The nonsense variant (p.R339*) results in a change from Arg339 to a premature stop codon, indicating a loss-of-function protein.

### 3.3. CCDC7 Spatial and Temporal Expression Patterns in the Human Placenta

**Figure 3:**
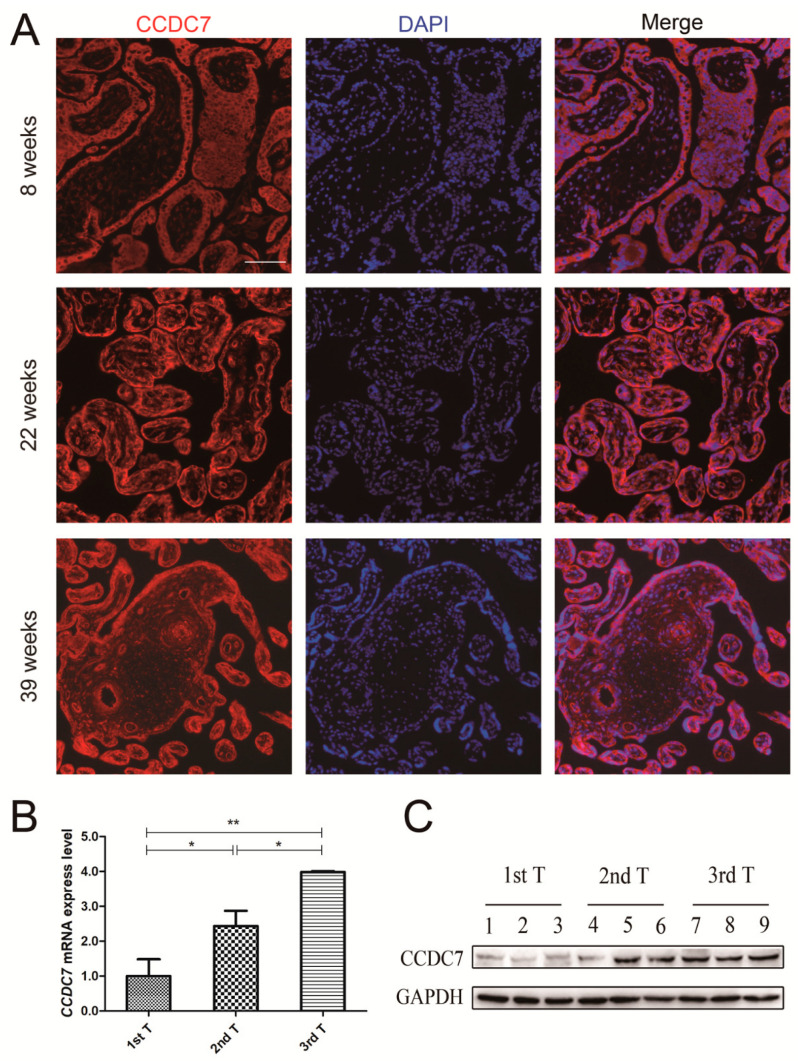
CCDC7 localization and expression in human placenta of different trimesters. (**A**) Immunofluorescence showed that CCDC7 localized in cytoplasm of cytotrophoblast, syncytiotrophoblast, and vascular epithelial cells in the human placenta and did not change during gestation; quantitative real-time polymerase chain reaction (**B**) and Western blotting (**C**) showed that the mRNA and protein levels of CCDC7 elevated with advancing gestational age. * *p* < 0.05, ** *p* < 0.01. Scale bars, 25 μm.

To determine where CCDC7 is localized in the human placenta, we performed immunofluorescence studies on placenta tissue sections. The localization of CCDC7 within the chorionic villi structure was examined via immunofluorescence on placenta tissue from each trimester across gestation. The general localization of CCDC7 in representative samples from early (8 weeks), middle (22 weeks), and late (39 weeks) gestation is provided in Figure 3A. During the first trimester, CCDC7 was detected in syncytiotrophoblast cells, cytotrophoblast cells, and trophoblast columns. In the second and third trimesters, intense staining was still found in syncytiotrophoblast cells and cytotrophoblast cells. 

In order to determine whether CCDC7 expression changes over gestation, we explored the mRNA and protein levels of CCDC7 in placental tissues at specific gestational time windows. As depicted in Figure 3B, a significant increase in CCDC7/GAPDH mRNA expression was found in placental tissues with advanced gestational age. Western blotting further confirmed that the expression of CCDC7 protein increased as pregnancy advanced (Figure 3C). These observations suggest that CCDC7 plays a key role in healthy placental development and function. The specific expression of CCDC7 in placental tissue indicated that CCDC7 plays an essential role in maintaining gestation.

### 3.4. Expression and Distribution of Rare CCDC7 Variants in the Placenta

**Figure 4:**
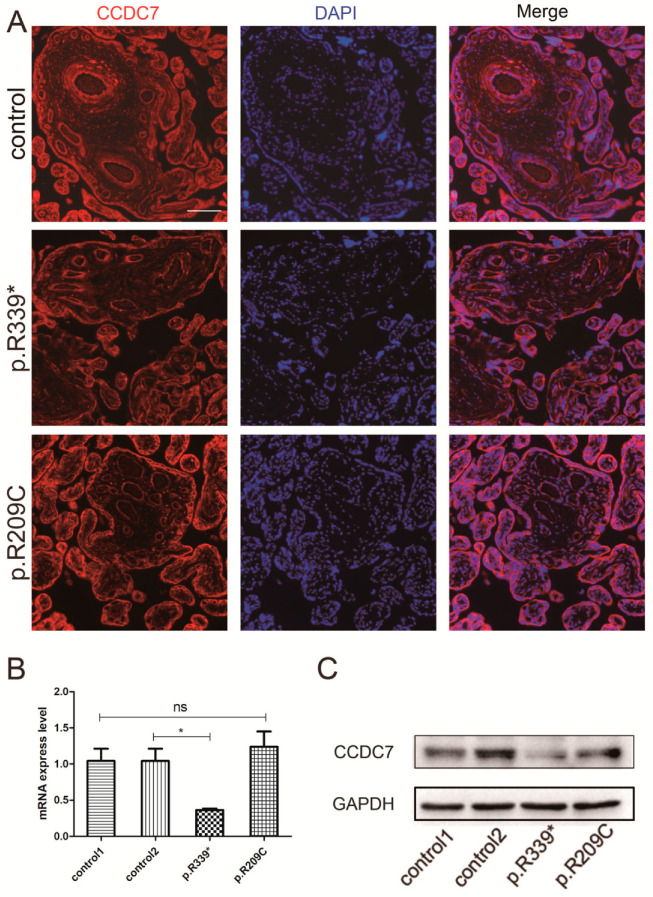
CCDC7 localization and expression in placenta from preeclampsia families and controls. (**A**) Immunofluorescence shows that the localization of CCDC7 in the placenta from the two preeclampsia families was the same as that in the controls. Quantitative real-time PCR (**B**) and Western blotting (**C**) showed that mRNA and protein levels of CCDC7 were decreased in the placenta from family B (p.R339*) and did not change in the placenta from family A (p.R209C) compared with those of age-matched controls (p.R339* vs. control2; p.R209C vs. control1). * *p* < 0.05; ns, not significant. Scale bars, 25 μm.

To investigate whether these variants detected in our study affected CCDC7 localization and expression, we compared CCDC7 localization and expression in the placentas from two preeclampsia families with age-matched controls. As showed by immunofluorescence, the localization of CCDC7 in the placenta did not alter in either of the two families compared with that in the controls (Figure 4A). The results of RT-qPCR and Western blotting revealed that the expression level of CCDC7 was significantly decreased in the p.R339* variant placenta compared with that in the controls. And, CCDC7 mRNA and protein expression showed little changes in the p.R209C variant placenta (Figure 4B,C). Collectively, these results imply that CCDC7 is involved in the pathology of preeclampsia.

### 3.5. Effects of CCDC7 on JEG-3 Cell Migration and Invasion

**Figure 5:**
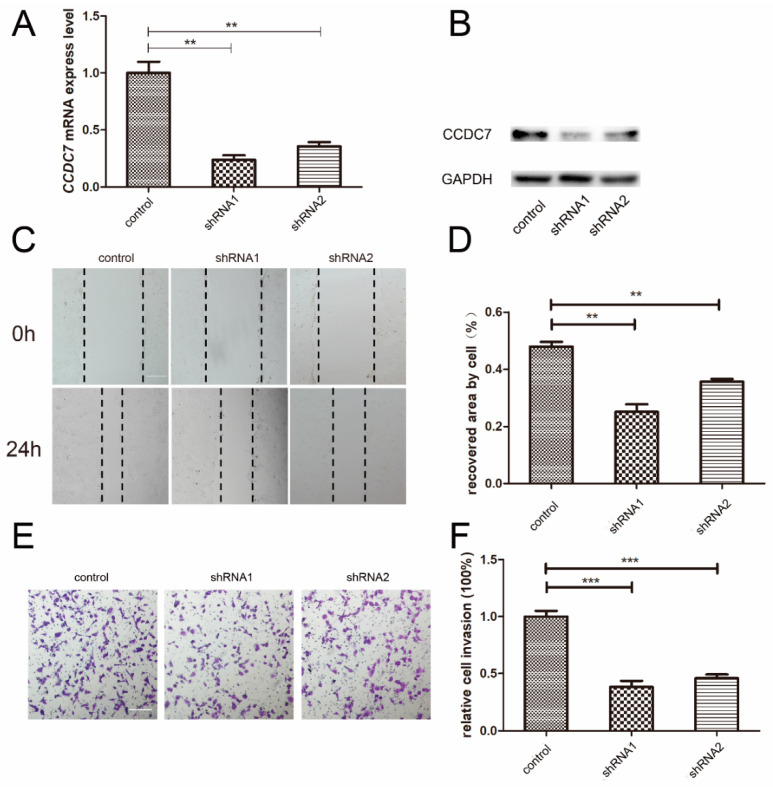
Effects of CCDC7 knockdown on the proliferation, migration, and invasion of JEG3 cells. Quantitative real-time PCR (**A**) and Western blotting (**B**) showed that shRNA1 and shRNA2 significantly decreased the expression level of CCDC7 mRNA and protein. Wound-healing assay (**C**,**D**) and transwell invasion assay (**E**,**F**) showed that the migration and invasion abilities of trophoblast cells decreased after knockdown of CCDC7. ** *p* < 0.01, *** *p* < 0.001. Scale bars, 50 μm in (**C**,**E**).

The aberrant migration and invasion abilities of extravillous trophoblasts (EVTs) have been discovered to be involved in the pathogenesis of PE. In this study, the stable transfection of shRNAs was used to silence *CCDC7* in choriocarcinoma JEG-3 cells. The results of RT-qPCR and Western blotting confirmed that the expression of CCDC7 was significantly decreased following transfection of the cells with shRNAs (Figure 5A,B). Scratch wound-healing and Matrigel invasion assays were conducted to investigate the effects of CCDC7 on the migration and invasion of trophoblast cells. As shown in Figure 5C–F, the migration and invasion capacities of JEG-3 cells were inhibited after *CCDC7* silencing. These findings indicate that abnormal CCDC7 expression might contribute to abnormal trophoblast migration and invasion. 

## 4. Discussion

In a previous study, the pathogenesis of preeclampsia was considered to involve two stages: abnormal placentation and the development of the maternal syndrome [20]. EOPE may arise due to defective placentation, while LOPE may center around interactions between the normal senescence of the placenta and a maternal genetic predisposition to cardiovascular and metabolic disease [21]. In this study, we detected two rare *CCDC7* variants in the preeclampsia offspring from two unrelated families and found that *CCDC7* had a crucial role in the development of preeclampsia by regulating the migration and invasion of trophoblast cells. This finding reveals the potential importance of rare variants in EOPE pathogenesis and supports the relationship between EOPE and abnormal placentation caused by genetic variation.

Complex disorders are often considered to be polygenic, with many common variants, each with modest effects that contribute to disease risk [7]. Importantly, the common variants detected to be responsible for preeclampsia have rarely been duplicated in different groups. By contrast, familial cases occurred commonly in preeclampsia. However, the probability is very low that a great number of common variants can be inherited by different offspring in one family to reach a threshold that causes a phenotype according to the law of segregation. Thus, rare variants are speculated to play a role in preeclampsia, especially familial preeclampsia, since rare variants often have greater effects than common variants, and it is more likely that a few rare variants are inherited by different offspring to reach the threshold. Recent studies have reported that rare variants contribute to preeclampsia. In women from multiplex preeclampsia families, Melton et al. [22] found a significant association between two relatively rare missense variants in *C1orf35* and *QRFPR* and preeclampsia. The rare variant rs71324987 in *MST1* identified in these families might also be associated with preeclampsia based on functional annotation and in silico prediction [23]. In our study, we found that rare variants of *CCDC7* disturbed the migration and invasion of trophoblast cells and contributed to the development of preeclampsia, which further indicates a role of rare variants in the offspring of mothers with preeclampsia.

Coiled-coil domain-containing (CCDC) is a structural motif that had been identified in proteins and is involved in diverse biological processes [24]. Previous studies discovered that genes containing this motif play a role in many malignant tumors. For example, CCDC3 was found to be a nuclear tumor suppressor in breast cancers [25], and CCDC60 is a potential marker correlated with the prognosis of head and neck squamous cell carcinoma [26]. The *CCDC7* gene was associated with the development of human cervical cancer [27] and colorectal cancer [28]. However, the function of CCDC7 remains unclear so far. Although cancer and placenta development are two different events, some properties and molecular pathways are shared by them, such as the ability to invade healthy tissues and the activation of VEGF to promote sustained angiogenesis [29]. Previous studies revealed that several CCDC members such as CCDC3 and CCDC85B exert a role in the migratory and invasive abilities of multiple cancer cells [30,31]. In this study, we detected two rare variants of CCDC7 in the offspring of two preeclampsia families and found that the knockdown of *CCDC7* impaired the migration and invasion of trophoblast cell. Additionally, many CCDC proteins are involved in vascular stabilization and inflammation. CCDC97 and CCDC107 were found to be associated with coronary artery disease and diabetes-associated atherogenesis, respectively [32,33]. CCDC22 affects the proinflammatory response via the downregulation of NF-κB signaling [34]. CCDC134 promotes T-cell activation through the regulation of early T-cell receptor signaling [35]. Angiogenic imbalance and abnormal inflammatory are believed to underlie the pathogenesis of severe preeclampsia particularly maternal syndrome [21]. The decreased CCDC7 level may contribute to the maternal syndrome by dysregulating these cytokines. These findings could contribute to the further understanding of the role of CCDC7 in cancer and placental development associated with preeclampsia.

Although our study revealed a potential role of rare offspring variants in preeclampsia, there are some limitations. First, our study focused on rare variants of preeclampsia offspring, and both of these two rare variants of *CCDC7* were maternal. Therefore, further study may be needed on the role of rare variants of *CCDC7* on the maternal syndrome, although preeclampsia did not occur in all pregnancies of women carrying rare variants of *CCDC7*. Second, the details of the molecular mechanism by which the downregulation of CCDC7 decreased the migration and invasion ability of trophoblast cells remain unclear. Further investigations on the mechanism are needed for a better understanding of the function of CCDC7 and the molecular mechanism of preeclampsia caused by loss-of-function mutations in the *CCDC7* gene. Additionally, the sample size was small in this study: two Han families. Rare variants in the *CCDC7* gene screened in more families, especially of different races, are of importance for investigating the role of rare CCDC7 variants in the general population.

## 5. Conclusions

In this study, we detected rare loss-of-function variants of *CCDC7* in two EOPE families, one of which resulted in decreased expressions of *CCDC7* mRNA and protein. Further testing revealed that the downregulation of CCDC7 destroyed the migration and invasion abilities of trophoblast cells. These findings indicate that CCDC7plays a crucial role in EOPE via regulating the migration and invasion of trophoblast cells.

## Figures and Tables

**Table 1:** Detail features of mothers and offspring from preeclampsia families.

Family No.	A	A	A	B	B	
Mother	II-2	II-6	II-8	I-2	II-2	
Onset time at pregnancy (Week)	24^+1^	27^+0^	32^+0^	NA	32^+1^	
Gestational age at delivery (Week)	34^+4^	27^+4^	32^+0^	36^+4^	36^+4^	
High blood pressure *	Yes	Yes	Yes	Yes	Yes	
Proteinuria *	Yes	Yes	Yes	Yes	Yes	
Impaired liver function *	No	No	No	NA	No	
Renal insufficiency *	No	No	No	NA	No	
Thrombocytopenia *	No	No	No	NA	No	
Pulmonary edema *	No	No	No	NA	No	
Headache and/or visual symptoms *	No	No	Yes	NA	No	
Offspring	III-1	III-8	III-9	II-2	III-2	IIII-3
Sex	Female	Female	Female	Female	Male	Female
Birth weight (g)	2100	750	1500	NA	2340	2470
Apgar score 1 min	10′	8′	4′	NA	10′	10′
Apgar score 5 min	10′	10′	7′	NA	10′	10′
Apgar score 10 min	10′	10′	10′	NA	10′	10′

* Diagnostic criteria for all these symptoms were referred from the ACOG practice bulletin No. 202.

## Data Availability

The data presented in this study are available on request from the corresponding author.

## Supplementary Materials

The following supporting information can be downloaded at: https://www.mdpi.com/article/10.3390/jpm14030253/s1. More detail information about samples and DNA extraction, maternal DNA contamination analysis, and Whole exome sequencing.

## Abbreviations

CCDC	Coiled-coil domain-containing
CTBs	cytotrophoblast cells
EOPE	Early-onset preeclampsia
GWAS	Genome-wide association studies
LOPE	Late-onset preeclampsia
MMPs	Matrix metalloproteinases
PVDF	Polyvinylidene fluoride
qRT-PCR	Quantitative real-time polymerase chain reaction
SDS-PAGE	Sodium dodecyl sulfate-polyacrylamide gel-electrophoresis
STBs	Syncytiotrophoblasts
WES	Whole-exome sequencing
WGS	Whole-genome sequencing
