# Supplementary material for: Rare Mutations in CCDC7 Contribute to Early-Onset Preeclampsia by Inhibiting Trophoblast Migration and Invasion
Source: J Pers Med. 2024 Feb 27;14(3):253. doi: 10.3390/jpm14030253 (PMC10971223; doi:10.3390/jpm14030253)
Supplement: Supplementary file 1 [file jpm-14-00253-s001.zip › jpm-2846142-supplementary.pdf]

Samples and DNA extraction

The peripheral blood (5-10ml) was collected from women with preeclampsia and their relatives except subjects III-8 in family A and III-2 and III-3 in family B. In these three subjects, the cord blood (5-10ml) was collected instead of peripheral blood in order to avoid the damage of blood drawing when they were born. Genomic DNA was extracted from these samples by using the DNeasy Blood and Tissue Kit (Qiagen, Hilden, Germany) according to the manufacturer's standard procedure.

Maternal DNA contamination analysis

Multiple QF-PCRs were performed on short tandem repeat (STR) sites in chromosomes 21, 18, 13, X, and Y. Chromosome 21 included D21S1435, D21S11, D21S1411, D21S1444, D21S1442, and D21S1437. Chromosome 18 included D18S978, D18S535, D18S386, D18S976, and GATA178F11. Chromosome 13 included D13S742, D13S634, D13S628, D13S305, and D13S1492. The sex loci included SRY, ZFYX, T1, T3, DXS1187, XHPRT, DXS2390, DXYS267, DXYS218, and AMELXY. The amplified product was subjected to capillary electrophoresis using an ABI-3130XL Genetic Analyzer. Maternal DNA contamination was excluded in the cord blood from subjects III-8 in family A and III-2 and III-3 in family B.

Whole exome sequencing

WES was conducted in all cases using next-generation sequencing platforms in the iGeneTech Co. Ltd (Beijing, China). Sequenced reads were aligned to human reference genome GRCh37/hg19. Software including Samtools and GATK were used to call, sort and index variants from aligned sequence files. Annotation was performed using ANNOVAR. Detailed parameters were shown in Table SI. The raw bases was 18413.75 ± 3493.56 Mb and QC rate was 96.48 ± 0.84%. Target mean depth was 140.60 ± 23.60 and 10 × coverage rate was 99.06 ± 0.35%. Thus, WES data was sufficient for the following analysis.

Table SI WES parameters in cases from family A and family B

Sequencing metrics	Mean ± standard deviation
Raw bases (Mb)	18413.75 ± 3493.56
Clean bases (Mb)	17762.72 ± 3359.90
QC rate (%)	96.48 ± 0.84
Average read length	145.07 ± 2.40
Mapped reads (M)	109.83 ± 28.52
Target covered size	62843941 ± 62942
Target mean depth	140.60 ± 23.60
Coverage rate (%)	99.78 ± 0.10
4×coverage rate (%)	99.59 ± 0.14
10×coverage rate (%)	99.06 ± 0.35
20×coverage rate (%)	97.54 ± 1.09
